# Crosstalk between NUAK1 and the SETD1A complex regulates glycolysis and gluconeogenesis upon glucose deprivation

**DOI:** 10.1016/j.jbc.2026.113598

**Published:** 2026-09-23

**Authors:** Zhen Wang, Yan Hao, Shiyuan Chen, Laurence Florens, Jerry L. Workman, Tamaki Suganuma

**Affiliations:** 1 Workman Lab, Stowers Institute for Medical Research, Kansas City, Missouri, USA; 2 Systems Mass Spectrometry, Stowers Institute for Medical Research, Kansas City, Missouri, USA; 3 Computational Biology, Stowers Institute for Medical Research, Kansas City, Missouri, USA

**Keywords:** NUAK1, glucose depletion, SETD1A complex, gene expression, histone H3pT11

## Abstract

Glucose is a crucial energy source, and glucose metabolism represents central chemical reactions in redox homeostasis. We previously found that histone H3 threonine 11 phosphorylation, which requires histone H3 lysine 4 trimethylation, promotes the transcription of genes regulating mitochondrial respiratory pathways during limited glucose in yeast. Here, we discovered that global levels of histone H3 threonine 11 phosphorylation increase during glucose depletion in human cells. The human NUAK1 kinase phosphorylates H3T11 and promotes expression of the genes regulating the p53-dependent DNA damage response upon glucose depletion. Remarkably, NUAK1 associates with the SETD1A histone H3K4 methyltransferase complex. Upon glucose depletion NUAK1 and the SETD1A complex promote expression of *HK2* and *PCK2*, which catalyze the first rate-limiting steps of glycolysis and gluconeogenesis, respectively, in addition to genes regulating the mitochondrial respiratory pathway. In combination with constant histone H3 lysine 4 trimethylation levels during glucose depletion, NUAK1-mediated H3T11 phosphorylation may function as the anchor in the rewiring of gene expression during glucose depletion.

Glucose is an important carbon source for cellular energy and is primarily catabolized to pyruvate and ATP in glycolysis. Pyruvate is further utilized for acetyl-CoA synthesis in the trichloroacetic acid (TCA) cycle, generating ATP. Glucose deprivation induces mitochondrial oxidative phosphorylation and readily produces reactive oxygen species (ROS) (1, 2). Increased mitochondrial respiration upon glucose deprivation is typically observed in cancers (3). Misregulation of mitochondrial respiration and imbalanced redox systems also underlie neurodegenerative diseases (4).

Metabolites serve as key donors for posttranslational modifications of proteins including histones and influence gene expression (5, 6). Several metabolic enzymes also directly function in chromatin regulation and transcription (5). We previously found that yeast Pyk1, a homolog of human pyruvate kinase M2, is a component of the serine-responsive S-adenosyl-L-methionine (SAMe)-containing metabolic enzyme (SESAME) complex, which consists of serine metabolic enzymes (Ser33, Ser3, and Shm2), SAMe synthetases (Sam1 and Sam2), and an acetyl-CoA synthase (7). Pyk1 in SESAME phosphorylates histone H3T11 using phosphoenol pyruvate as a phosphate donor under growth in rich media and serves SAMe to the Set1 histone H3K4 methyltransferase complex. In addition, we found that H3K4me3 is required for the histone H3 threonine 11 phosphorylation (H3pT11) *in vitro* and *in vivo* (7). Therefore, the association of SESAME with the Set1 complex establishes crosstalk between H3K4me3 and H3pT11 in response to glycolysis and glucose-derived serine metabolism (7).

Upon nutritional stress in yeast H3T11 phosphorylation is carried out by the activated Tda1 kinase (8). Phosphorylation of H3T11 by Tda1 is required for the metabolic transition to respiration by switching H3pT11 deposition from fermentation regulatory genes to mitochondrial and stress responsive genes in yeast (8, 9). Notably, loss of H3pT11 alters the nutritional stress response at an early stage of chronological lifespan, leading to longevity in yeast (9). The human ortholog of yeast Tda1, AMPK-related serine/threonine kinase (NUAK1), has been shown to be involved in glycolysis and the oxidative stress response (10, 11, 12, 13). In addition, NUAK1 promotes Akt signaling by phosphorylation of Akt (Ser473) while NUAK1 controls subcellular compartmentalization of mTORC2 in growth factor-dependent manner (11). However, it is largely unknown how NUAK1 regulates gene expression.

There are six family members of the SET1 complexes in humans: SETD1A, SETD1B, KMT2A/MLL1, KMT2B/MLL2, KMT2C/MLL3, and KMT2D/MLL4 (14). They share core subunits DPY30, RBBP5, WDR5, and ASH2L. HCFC1 is a common subunit in the SETD1A, SETD1B, MLL1, and MLL2 complexes. CXXC1 and WDR82 are contained only in the SETD1A and SETD1B complex (14). The human SET1 complex consists of SETD1A, HCFC1, CXXC1, KMT2A, WDR5, ASH2L, RBBP5, WDR82, SETD1B, DPY30, and KMT2B. The SET1 complex family regulates transcription through histone H3K4 methylation at gene promoters or enhancers (15). Importantly, CXXC1 is a specific subunit in the SETD1A/SETD1B complex, and its yeast homolog Spp1 is required for H3K4me3 that is essential for H3pT11 (7, 16). Since histone H3K4me3 is required for the H3pT11 (7), CXXC1 is a key player for the crosstalk between H3K4me3 and H3pT11.

Here, we studied how NUAK1 responds to glucose deprivation, its relationship to human SET1 complexes, and its potential roles in metabolic processes.

## Results

### Interaction of NUAK1 with the SETD1A complex and p53 are facilitated upon glucose depletion

To find the biological roles of NUAK1 in response to the glucose availability, we purified Flag-tagged NUAK1 stably expressed in the nucleus of HEK293 cells cultured in normal Dulbecco’s modified Eagle’s medium (DMEM) medium containing 25 mM D-glucose and 1 mM sodium pyruvate or glucose- and pyruvate-free DMEM (here after referred to as “absence of glucose”) and analyzed the NUAK1 interacting proteins by mass spectrometry (Fig. 1, *A* and *B*). We found that all components of the SET1 complexes including SETD1A, HCFC1, CXXC1, KMT2A, WDR5, ASH2L, RBBP5, WDR82, SETD1B, DPY30, and KMT2B, were copurified with NUAK1 in both conditions (Fig. 1*B*). To confirm the mass spectrometry data, we further examined the copurified subunits of the SETD1A complex by Western blotting. SETD1A, CXXC1, and DPY30 were indeed associated with FLAG-purified NUAK1 (Fig. 1*C*). Interestingly, the SETD1A expression level was increased in the cells cultured in the absence of glucose (Fig. 1*C*, Input). We next proved these interactions at endogenous levels of expression in the nucleus of HEK293 cells by immunoprecipitation of endogenous NUAK1. The subunits of the SETD1A complex were associated with NUAK1 in the cells regardless of presence or absence of glucose (Fig. 1*D*). However, the expression levels of these subunits of the SETD1A complex, especially SETD1A, in immunoprecipitated NUAK1 were clearly increased in the cells cultured under glucose depletion compared to the presence of glucose (Fig. 1*D*, lanes 4 *versus* 3). Reciprocal immunoprecipitation with SETD1A or CXXC1 antibodies consistently revealed their interactions with NUAK1 and increased those interactions in the absence of glucose (Fig. 1*E*). Hence, the interactions of NUAK1 with the SETD1A complex were facilitated upon glucose depletion.

**Figure 1:**
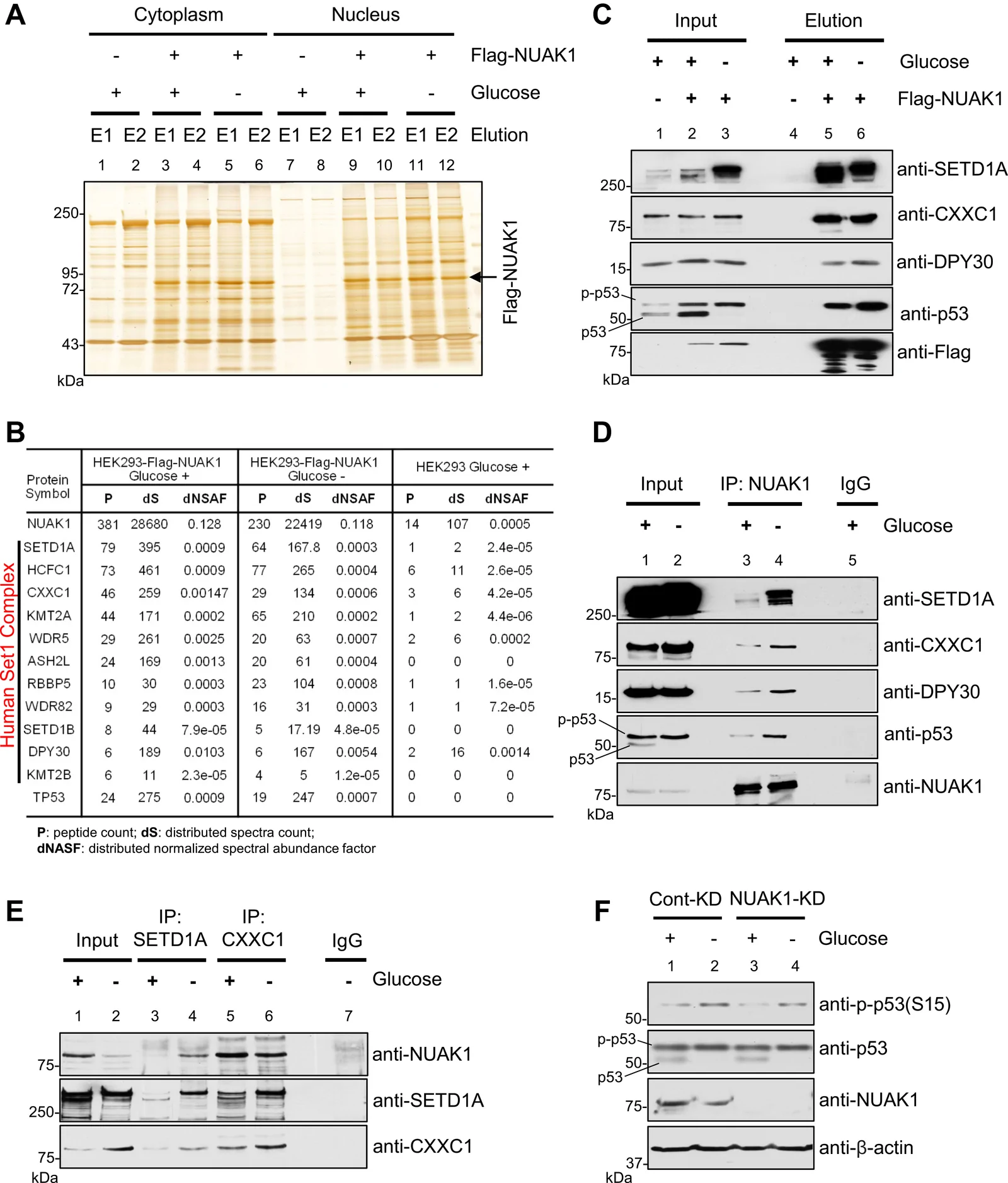
**NUAK1 interacts with SETD1A complex and p53.***A*, *silver staining* shows Flag-purified NUAK1 from cytoplasm and nuclear extracts of Flag-NUAK1 stably expressed HEK293 (+) or parental HEK293 (−) (control) cells cultured in the presenceor absence of glucose for 36 h. *B*, table displays representative proteins copurified with nuclear Flag-NUAK1 or controls by mass spectrometry analysis (*A*). *C*, input nuclear extracts for Flag purification, and elution of Flag-purified NUAK1 (+) and controls (−) was analyzed by Western blots. p-p53 (S15) indicates phosphor-p53 (serine 15) (*D*). *D* and *E*, immunoprecipitated (IP) endogenously expressed NUAK1 (*D*), SETD1A (*E*), and CXXC (*E*) in nuclear extracts from HEK293 were analyzed by Western blotting. IgG immunoprecipitants were used as controls. *F*, nuclear extracts from HEK293 cells cultured in the presence or absence of glucose for 36 h were examined by Western blots. Beta-actin was used as the loading control.

We also found that p53 copurified with NUAK1 by mass spectrometry (Fig. 1*B*). We observed these interactions at endogenously expressed levels (Fig. 1*C*). Previous studies suggested that NUAK1 and ATM kinase phosphorylate p53 serine 15 during glucose starvation (17). We found that the levels of p53 serine 15 phosphorylation (p-p53(S15)) were increased upon glucose depletion; however, the phosphorylation was not clearly reduced in NUAK1-KD cells compared to controls, suggesting that NUAK1 was not a primary kinase for p-p53(S15) (Fig. 1*F*). Importantly, only phosphorylated p53 (p-p53) interacted with NUAK1 and the SETD1A complex, and these interactions were increased upon glucose depletion (Fig. 1, *C* and *D*). Thus, NUAK1 and the SETD1A complex interact with p53 when p53 is phosphorylated upon glucose depletion.

### NUAK1 regulates gene expression involved in the p53 response to glucose depletion

We asked whether the role of NUAK1 led to changes in gene expression in response to glucose depletion. We first examined the effects of glucose depletion on gene expression in controls by RNA-sequencing. We found that the expression levels of genes regulating lipid metabolism: *LIPH* and *AKR1C3*, and genes associating with JNK signaling: *SH3RF2*, were significantly decreased upon glucose depletion (18, 19, 20) (Fig. S1). In contrast, genes regulating mitochondrial functions: *SHMT2*, *MTHF SESN2D2*, and *PCK2*, and genes encoding proteins associating with p53 (during DNA damage responses and p53 associating with mTOR): *SESN2* and *NUPR1*, were increased upon glucose depletion (21, 22, 23, 24, 25) (Fig. S1).

To understand the relevance of NUAK1 in changing gene expression levels upon glucose depletion, we compared genes with transcript levels between NUAK1-KD cells and controls cultured in the absence of glucose (Fig. 2*A*, y-axis); or between presence and absence of glucose in controls (Fig. 2*A*, x-axis). We then analyzed these correlations (Fig. 2*A*). Interestingly, there was a modest negative correlation between genes with changed expression levels upon glucose depletion in controls (x-axis) and genes with changed expression levels upon NUAK1 knockdown under glucose depletion (y-axis) (Fig. 2*A*). Thus, upregulated genes by glucose depletion in controls tend to be downregulated in NUAK1-KD cells cultured in the absence of glucose, and *vice versa*.

**Figure 2:**
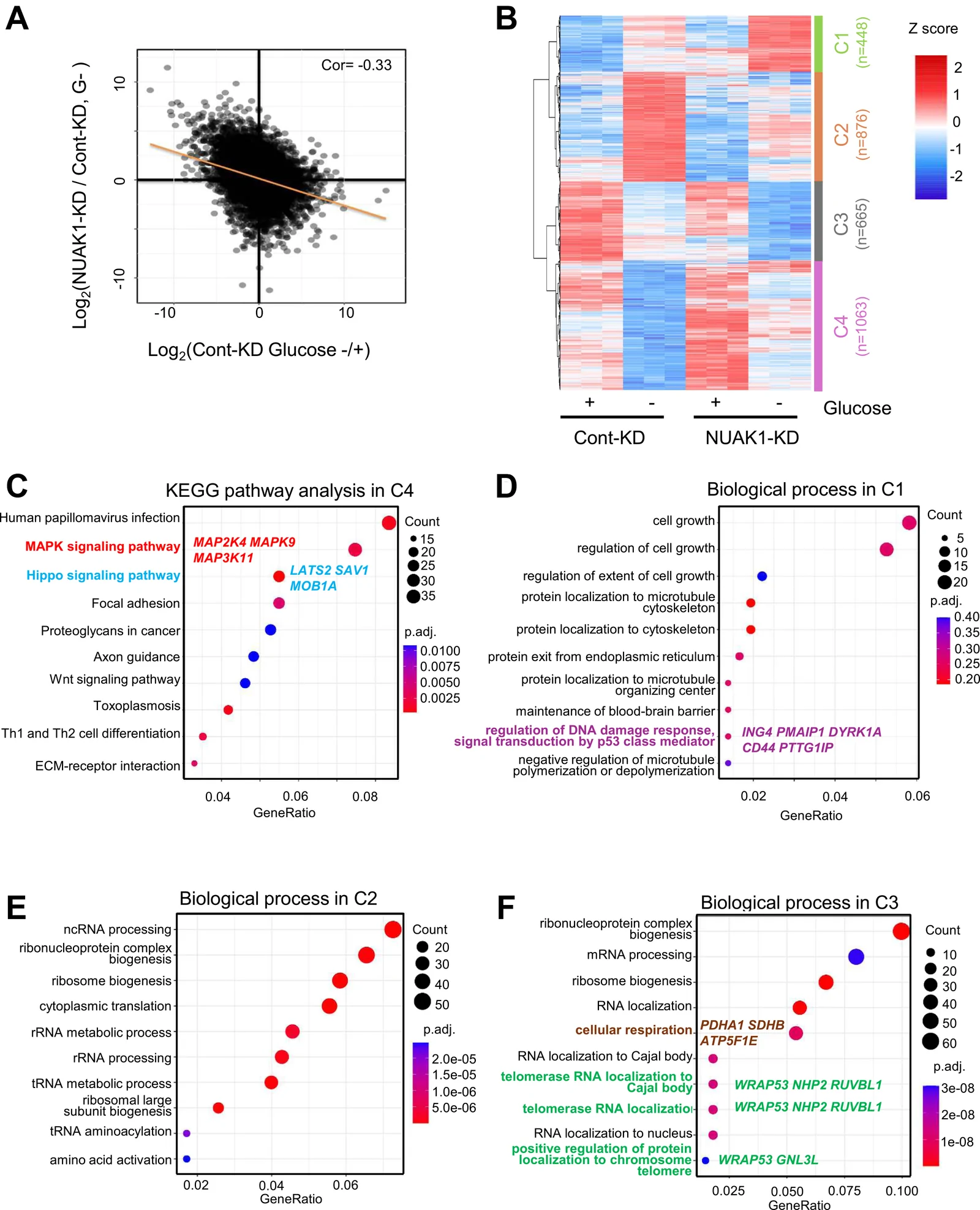
**NUAK1 regulates gene expression in response to glucose depletion.***A*, scatter plot displays a correlation between the genes with transcript levels compared between NUAK1-KD HEK293 cells and control-KD HEK293 cells cultured in the absence of glucose (y-axis); or between presence and absence of glucose in control-KD cells (x-axis) using RNA-sequencing (a correlation coefficient of −0.33). *B*, heatmap displays genes with transcript levels (between glucose (+) *versus* glucose (−) in control-KD or NUAK1-KD) compared between NUAK1-KD cells and control-KD cells cultured in the presence (+) or absence (−) of glucose by differentially expressed genes (DEGs) analysis using RNA-sequencing. The transcript levels are further clustered to four groups (C1-C4). n: numbers of genes in each cluster. *C*–*F*, dot plots display GO analysis clustered by KEGG pathway analysis (*C*) or biological process (*D–F*) for genes with changed transcript levels corresponding to clusters in Figure 2*B*. The top 10 processes based on the lowest adjusted *p*-value are listed (Table S1). GO, Gene Ontology; KEGG, Kyoto Encyclopedia of Genes and Genomes.

We then analyzed differentially expressed genes in NUAK1-KD cells *versus* controls cultured in the presence or absence of glucose with clustering to four groups (C1–C4) (Fig. 2*B*) and performed the Gene Ontology (GO) analysis for each cluster. We found that in cluster 4 expression levels of genes regulating MAPK signaling and Hippo signaling pathways were decreased upon glucose depletion in controls; however, expression levels of those genes were elevated in the presence and absence of glucose in NUAK1-knockdown cells compared to controls in each condition (Fig. 2, *B* and *C* and Table S1). In contrast, in cluster 1 upregulated genes upon glucose depletion in controls were further facilitated in NUAK1-KD upon glucose depletion (Fig. 2*B*). These genes included DNA damage responsive genes regulated by p53 (Fig. 2*D* and Table S1). Thus, loss of NUAK1 may harm the cells upon glucose depletion and induce p53 dependent DNA damage. There is also the possibility that NUAK1 suppresses p53 dependent transcription in DNA damage signaling upon absence of glucose.

Expression levels of genes in cluster 2 clearly increased upon glucose depletion in controls but those increases were attenuated in NUAK1-KD, indicating that the increased expression of these genes by glucose depletion was NUAK1 dependent. These genes function in ribosome biogenesis and translation (Fig. 2*E* and Table S1). Interestingly, cluster 2 included the genes regulating pyruvate metabolic process and NAD metabolic process, including *phosphoenolpyruvate carboxykinase 2, mitochondrial* and hexose catabolic process and monosaccharide metabolic processes including *hexokinase 2* (Table S1). HKs phosphorylate glucose to generate glucose-6-phosphate in the first step of glycolysis (26). PCK2 catalyzes the conversion of oxaloacetate to phosphoenol pyruvate in the first rate-limiting step of gluconeogenesis and suppresses ROS generation by controlling mitochondrial respiration during glucose starvation (3, 27). Thus, NUAK1 promotes genes regulating both glycogenesis and gluconeogenesis.

In cluster 3, the down regulated genes functioning in telomere synthesis upon glucose depletion in controls include *p53 WD repeat containing antisense to TP53*, which promotes p53 mRNA levels and sensitivity of p53-dependent apoptosis (28, 29). In addition, the down regulated genes upon glucose depletion in controls include mitochondrial genes, such as *pyruvate dehydrogenase E1 subunit alpha 1*, downregulation of which has been shown to lead to overproduction of lactate and ROS generation (30). Oxidative phosphorylation in mitochondria induced upon glucose depletion increases ROS generation (2). Notably, these cluster 3 genes were further suppressed upon glucose depletion in the NUAK1-KD (Fig. 2, *B* and *F* and Table S1). Therefore, we first measured ROS levels. The ROS levels increased upon glucose depletion in both control-KD cells and NUAK1-KD cells (Fig. S2, *A* and *B*). However, there were no significant differences in the ROS levels between control-KD and NUAK1-KD cells in either the presence or absence of glucose (Fig. S2, *A* and *B*). We next measured apoptotic cell populations. Apoptosis was substantially induced by NUAK1-KD compared to control-KD in the presence and absence of glucose although the apoptotic cells increased upon glucose depletion in both control-KD and NUAK1-KD (Fig. S2*C*). These data indicated that loss of NUAK1 induces apoptosis. Malfunctions of NUAK1 may also be a risk for ROS resistance.

### H3T11 phosphorylation by NUAK1 responds to glucose depletion in HEK293 cells

H3T11 is phosphorylated by Tda1 kinase upon nutrition stress in yeast (8). Through alignment of the protein sequences between *Saccharomyces cerevisiae* Tda1 and human NUAK1, we confirmed that both kinases share a kinase domain and disordered regions in their N terminus (Fig. 3*A*). Therefore, we examined whether human NUAK1, an ortholog of yeast Tda1, phosphorylates H3T11. We first examined the kinase activity of purified recombinant NUAK1 on recombinant histone octamers. We found that recombinant NUAK1 directly phosphorylates histone H3T11 (Fig. 3*B*).

**Figure 3:**
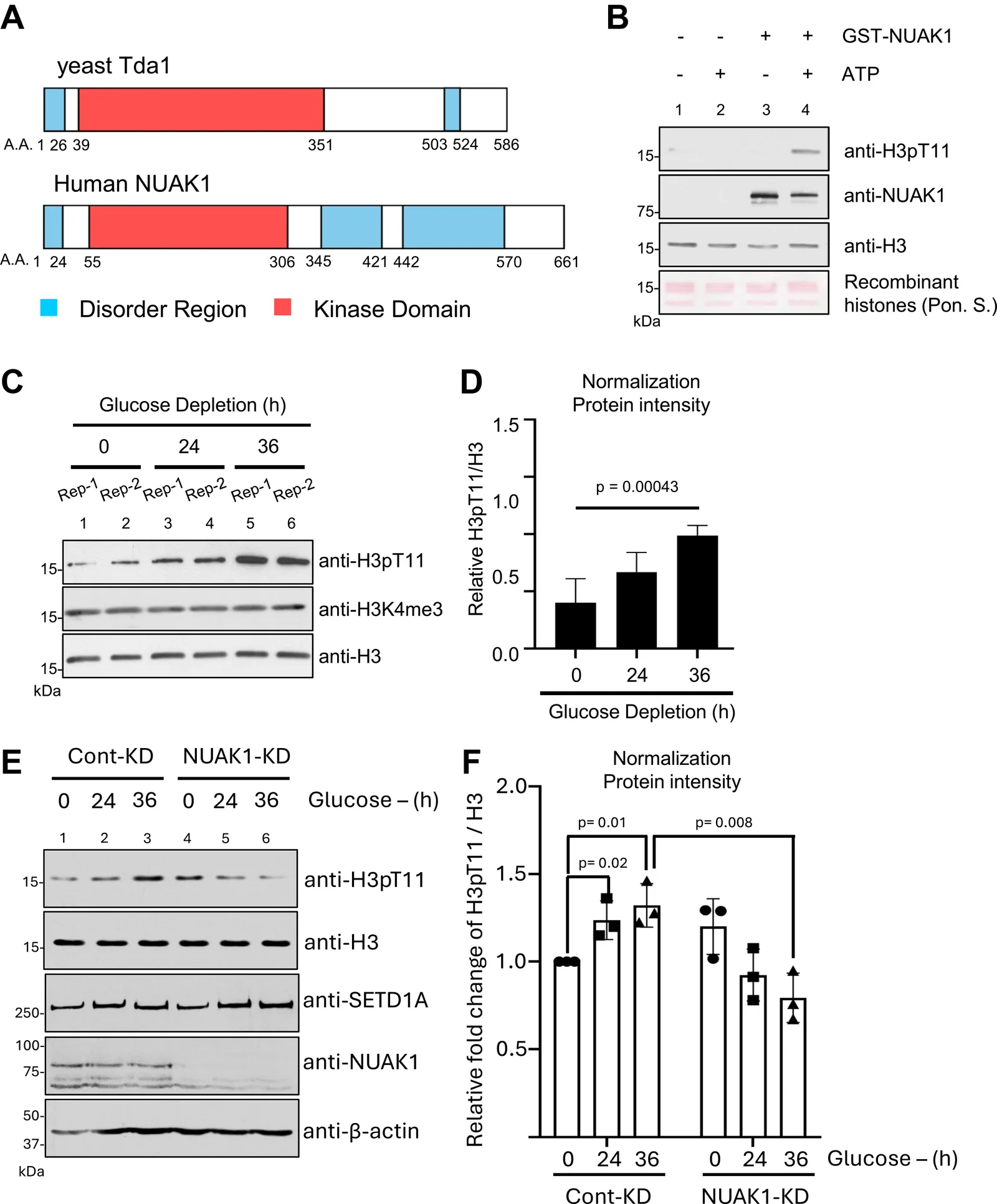
**NUAK1 phosphorylates H3T11 during glucose depletion.***A*, diagram of yeast Tda1 and human NUAK1. *B*, phosphorylation of purified recombinant GST-NUAK1 on recombinant histone octamers using 1 mM ATP was analyzed by Western blots. Histone H3 (H3) and ponceau staining (Pon. S) were used as the loading controls. *C*, histone extracts from HEK293 cells cultured in the presence or absence of 25 mM D-glucose and 1 mM sodium pyruvate for indicated time were examined by Western blots (*D*). Histone H3 (H3) was used as the loading control. Rep, repeat. *D*, bar graph displays normalization of H3pT11 signals compared to histone H3 signals in Western blots (*D*) (n = 5). *p*-values (P) were analyzed by comparisons between the normalized factors at 0-h point and 36-h point. *E*, nuclear extracts (for probing SETD1A, NUAK1, and β-actin) and histone extracts (for probing H3pT11 and H3) from NUAK1- and control-knockdown HEK293 cells cultured in the presence (0 h) or absence of glucose for 0, 24, and 36 h were examined by Western blots. Beta-actin and histone H3 (H3) were used as the loading controls. *F*, bar graph displays normalization of H3pT11 signals compared to histone H3 signals in Western blots (*E*) (n = 3). p, *p*-value. H3T11, histone H3 threonine 11.

To examine whether H3T11 phosphorylation responds to the availability of glucose, we cultured the HEK293 cells in the presence or absence of glucose for 0, 24, and 36 h. We extracted histones from chromatin pellets after cytoplasm and nuclear extracts were removed. We then analyzed the histone extracts by Western blotting. We found that the global levels of H3pT11 increased upon glucose depletion with their highest levels at 36 h’ of depletion (Fig. 3, *C* and *D*). However, there were no obvious differences in the levels of H3K4me3 in these conditions (Fig. 3*C*). Thus, the levels of H3T11 phosphorylation but not H3K4me3 responded to glucose depletion in human cells.

We next examined whether NUAK1 was responsible for increased H3pT11 upon glucose depletion. We analyzed H3pT11 levels in NUAK1- and control-knockdown cells cultured in the absence of glucose for 0, 24, and 36 h. While H3pT11 levels increased over time of glucose depletion in controls, H3pT11 levels decreased during glucose-depletion in NUAK1-KD cells (Fig. 3, *E* and *F*). Hence, these data indicated that H3pT11 responds to glucose depletion while H3K4me3 maintains steady levels, and that NUAK1 is a kinase for H3T11 phosphorylation under glucose depletion.

### NUAK1 and the SETD1A complex promote glycolysis upon glucose starvation

Previously we found that H3K4me3 is required for the H3pT11 (7). Therefore, we examined whether the interactions of the SETD1A complex with NUAK1 influence gene expressions in response to glucose depletion. We first analyzed the correlations of transcript levels compared between SETD1A-KD and controls cultured in the absence of glucose (Fig. 4*A* y-axis, Fig. S2) and between presence and absence of glucose in controls (Fig. 4*A* x-axis). We observed that the expression levels of genes responding to glucose depletion tended to be negatively correlated with those responding to SETD1A-KD in the absence of glucose, similar to the effects of NUAK1-KD (Figs. 2*A* and 4*A*). We clustered into four groups (C5-C8) in differentially expressed genes analysis of the transcripts compared between SETD1A-KD cells and controls cultured in the presence or absence of glucose (Fig. 4*B*). To find the pathways, which are coactivated by NUAK1 and the SETD1A complex in response to glucose depletion, we further compared transcript levels between NUAK1-KD, SETD1A-KD, and controls in the absence of glucose (Fig. 4*C*). We compared genes in three clusters: cluster 2 (Fig. 2*B*); which contains genes with increased expression levels in controls but whose induction was attenuated in NUAK1-KD, cluster 6 (Fig. 4*B*); which contains genes with increased expression levels in controls but whose induction was attenuated in SETD1A-KD, and cluster 10 (Fig. 4*C*); which contains genes expressed at lower levels in NUAK1-KD and SETD1A-KD cells compared to controls. Comparison of these three clusters showed that 124 genes were activated by NUAK1 and SETD1A during glucose depletion (Fig. 4*D*). We performed the GO analysis corresponding to 124 genes down regulated by the NUAK1-KD and/or SETD1A-KD upon glucose depletion (cluster 2 in Figure 2, cluster 6 in Fig. 4*B*, and cluster 10 in Fig. 4*C*) shown in 3venn diagram in Figure 4*D* (Fig. 4*E*). Notably, the expression of *HK2* was suggested to be commonly down-regulated in NUAK1- and SETD1A-KD cells (Fig. 4*E*, corresponding to Fig. 4*D* ‘124’, Table S2). In addition, expression of ER stress response genes, such as *activating transcription factor 3*, might be suppressed in NUAK1- and SETD1A-KD cells upon glucose depletion (Fig. 4*E*).

**Figure 4:**
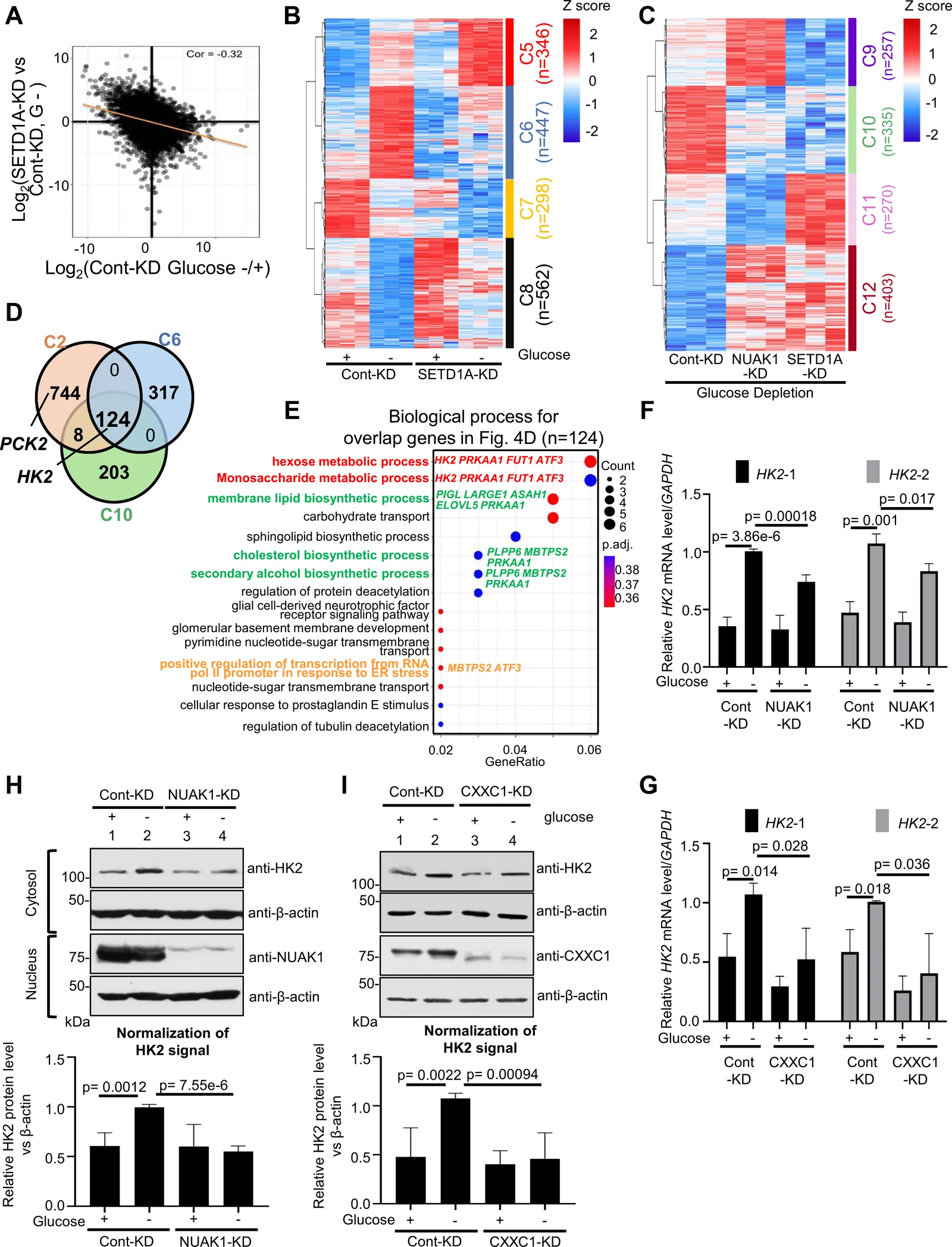
**NUAK1 and SETD1A complex promote glycolysis during glucose depletion.***A*, scatter plot displays a correlation between the genes with transcript levels compared between SETD1A-KD HEK293 cells and control-KD HEK293 cells cultured in the absence of glucose (y-axis); or between presence and absence of glucose in controls (x-axis) using RNA-sequencing (a correlation coefficient of −0.32). *B* and *C*, heatmaps show genes with transcript levels (*p*-value < 0.05 between glucose (+) *versus* glucose (−) in control (*B* and *C*), SETD1A-KD (*B* and *C*), or NUAK1-KD (*C*)) compared between SET1A-KD cells (*B* and *C*), NUAK1-KD cells (*C*), and control-KD cells (cultured in the presence (+) or absence (−) of glucose). *D*, Venn diagram shows numbers of genes with reduced transcripts in NUAK1-KD (cluster 2 in Fig. 2*B*), genes with reduced transcripts in SETD1A-KD (cluster 6 in Fig. 4*B*), and genes with reduced transcripts in both NUAK1-KD and SETD1A-KD upon glucose depletion (cluster C10 in Fig. 4*C*) measured by DEGs analysis. *E*, dot plots display Go analysis clustered by biological process for genes with reduced expressions in NUAK1-KD and SETD1A-KD upon glucose depletion corresponding to 124 genes in Figure 4*D*. The *top* 15 processes based on the *lowest* adjusted *p*-value are listed (Table S3). *F* and *G*, bar graphs display *HK2* mRNA level in NUAK1-KD (*F*), CXXC1-KD (*G*), and control-KD cells cultured in the presence (+) or absence (−) of glucose for 36 h analyzed by quantitative RT-qPCR (n = 3). p, *p*-value. *H* and *I*, cytoplasmic and nuclear extracts from NUAK1-KD cells (*H*), CXXC1-KD cells (*I*), and Control-KD cells cultured in the absence of glucose for 36 h were examined by Western blots (*upper panels* in *H* and *I*). Beta-actin was used as the loading controls (*upper panels* in *H* and *I*). Bar graphs in *bottom panels* display normalizations of HK2 signals compared to Beta-actin signals in Western blots (n = 5). p, *p*-values (P). DEG, differentially expressed genes; HK2, hexokinase 2; RT-qPCR, reverse transcription quantitative polymerase chain reaction.

We further examined the mRNA levels of *HK2* by quantitative RT-PCR and protein levels of HK2. Since yeast ortholog of the human CXXC1, Spp1, is required for H3K4me3 (7, 16), we compared *HK2* expression levels in NUAK1-KD, CXXC1-KD, and control cells cultured in the presence or absence of glucose. We found that *HK2* mRNA expression levels and protein levels were increased upon glucose depletion in controls (Fig. 4, *F–I*). However, this increased expression was reduced in NUAK1-KD and CXXC1-KD upon glucose depletion (Fig. 4, *F–I*). These data indicated that NUAK1 and the SETD1A complex are required for the HK2 expression and coregulate glycolysis in response to glucose depletion (Fig. 5*A*).

**Figure 5:**
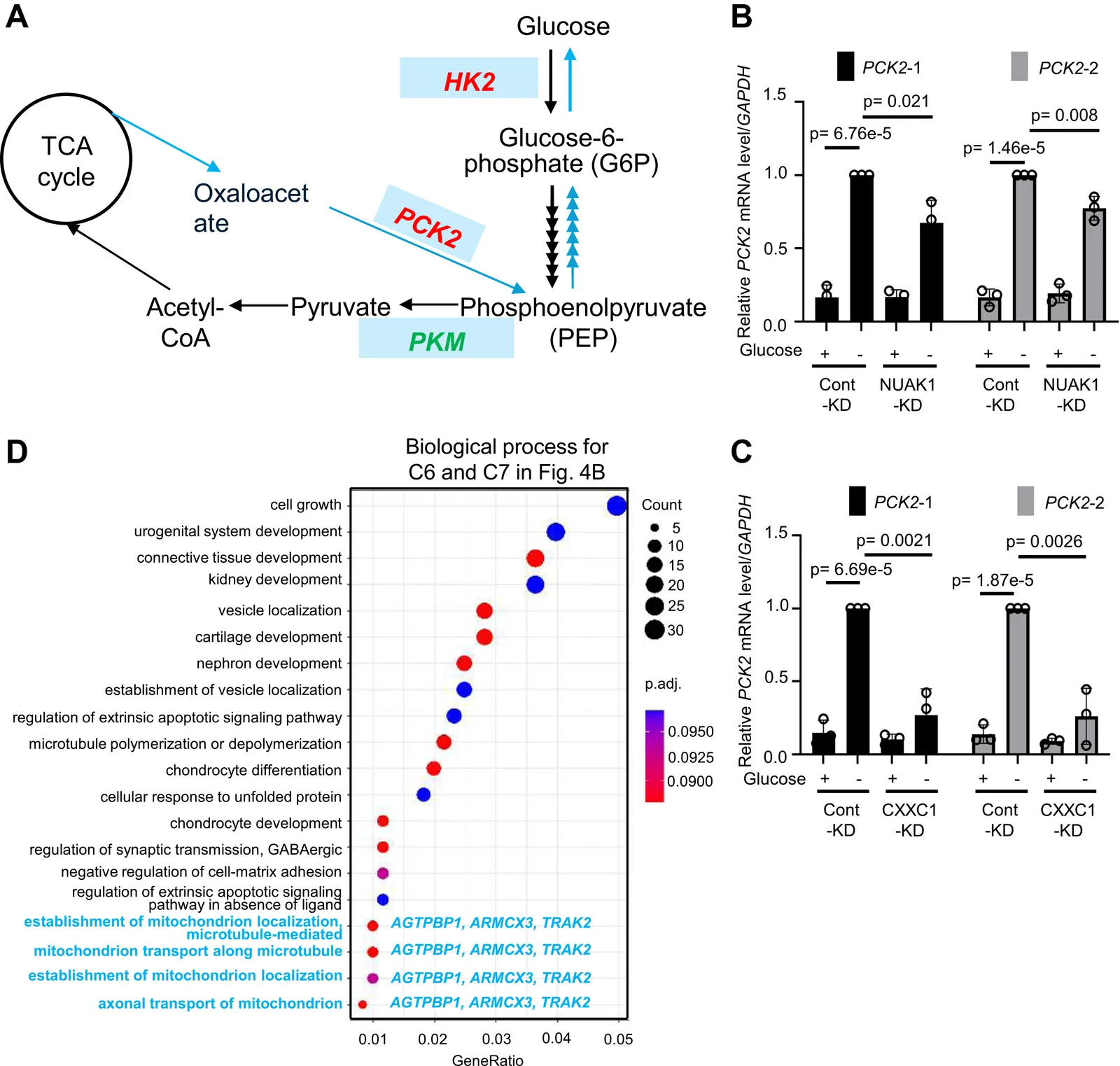
**NUAK1 and SETD1A promotes glycolysis and gluconeogenesis during glucose depletion.***A*, diagram of glycolysis (*black arrows*) and gluconeogenesis (*blue arrows*) associated with HK2 and PCK2. *B* and *C*, bar graphs display *PCK2* mRNA level in NUAK1-KD (*B*), CXXC1-KD (*C*), and control-KD cells cultured in the presence (+) or absence (−) of glucose for 36 h analyzed by quantitative RT-qPCR (n = 3). p, *p*-value. *D*, dot plot displays Go analysis clustered by biological process for genes with reduced expression in SETD1A-KD upon glucose depletion corresponding to cluster 6 and 7 in Figure 4*B*. The *top* 20 processes based on the *lowestp*-value are listed. HK2, hexokinase 2; PCK2, phosphoenolpyruvate carboxykinase 2.

We wondered whether the SETD1A complex contributed to the expression of gluconeogenesis gene, *PCK2*, which required NUAK1 upon glucose depletion (Fig. 2*B* cluster C2, Fig. 4*D* 744 genes, Fig. 5*A*, and Table S1) although it was not clustered in common downregulated 124 genes (Fig. 4*D*). Interestingly, upon glucose depletion mRNA levels of *PCK2* increased in controls were decreased in CXXC1-KD and NUAK1-KD although the reductions were lesser in NUAK1-KD compared to those in CXXC-KD (Fig. 5, *B* and *C*). In addition, expression levels of genes regulating mitochondrial function including *ATP/GTP binding carboxypeptidase 1*, *armadillo repeat containing X-linked 3*, and *trafficking kinesin protein 2*, tended to be decreased in SETD1A-KD compared to controls upon glucose depletion (31, 32, 33) (Fig. 5*D* and Table S3). Expression levels of *ATP/GTP binding carboxypeptidase 1* were also decreased in NUAK1-KD upon glucose depletion (Table S3). These data suggested that loss of NUAK1 and the SETD1A complex leads to changes in mitochondrial respiration and attenuation of gluconeogenesis.

Together, our study suggested the association of NUAK1 with the SETD1A complex plays crucial roles in rewiring glucose metabolism and respiration pathways in response to glucose depletion by promoting first steps of glycolysis and glycogenesis. However, suppressive effects of NUAK1-KD on the expression of genes regulating p53-dependent stress response and mitochondrial respiration were slightly moderated upon glucose starvation (Fig. 2*B* cluster C2). This suggests that loss of NUAK1 may cause tolerance to limited glucose as seen in nutrition stress resistance in histone H3T11A mutant in yeast (8).

## Discussion

Glycolysis is a catabolic process of glucose, whereas gluconeogenesis is an anabolic process synthesizing glucose. Our investigations show that NUAK1 and the SETD1A complex promote the expression of key enzymes, *HK2* and *PCK2*, in glycolysis and gluconeogenesis, respectively, during glucose depletion; however, these findings are seemingly contradictive. Previous study revealed that HK2-KD attenuates glucose flux to pyruvate and lactate while fluxes of TCA cycles are maintained, leading to elevation of oxidative phosphorylation and growth inhibition in Huh-7 human hepatoma cell line (34). The proliferation of HK2-KD cells dominantly depends on *de novo* synthesized serine (34), suggesting that one-carbon metabolism is also crucial for growth of HK2-KD cells (5). PCK2-KD increases the abundance and interconversion of TCA cycle intermediates, mitochondrial respiration, and glutathione oxidation under glucose and serum starvation in H23 Non-small cell lung cancer cell line (3). We found that the cell viability was slightly reduced upon glucose depletion in control-KD cells [viability: 93% in glucose (+) and 88% in glucose (−)] but was further reduced in NUAK1-KD cells regardless of the presence of glucose [84–85% in glucose (+) and 79% in glucose (−)]. In CXXC-KD cells, the cell viability was approximately 4% reduced upon glucose depletion [viability: 94% in glucose (+) and 90% in glucose (−)] (Fig. S4). Thus, NUAK1 is important for cellular fitness regardless of the presence or absence of glucose (Figs. S2*C* and S4). Loss of SETD1A (CXXC-KD) did not significantly influence cell survival (Fig. S4). Therefore, PCK2 protects cells from oxidative stress caused by nutritional depletion while it maintains gluconeogenesis (3). The common role between HK2 and PCK2 is maintaining cell survival through sensing the oxidative phosphorylation in mitochondria.

Importantly, NUAK1 histone H3pT11 kinase and the SETD1A H3K4 methyltransferase complex are required for the expression of HK2 and PCK2, strongly suggesting that initial steps of glycolysis and gluconeogenesis are transcriptionally regulated during glucose depletion. H3K4 methylation by SETD1A requires SAMe, which is an output of one-carbon metabolism (5). H3K4 methylation by SETD1A may be feedback to glycolysis to maintain cell growth during glucose depletion. In addition to NUAK1-dependent expression of p53-mediated DNA damage responsive genes, NUAK1 mediated H3pT11 may regulate transcriptions of genes to suppress ROS generation and oxidative damage by sensing availability of ATP, which is a phosphate donor for its phosphorylation and is mainly generated in mitochondria.

We found that global levels of H3K4me3 are steady; however, H3pT11 levels increase during glucose depletion. H3K4me3 is required for H3pT11 (7). Thus, the interactions of the SETD1A complex with NUAK1 connect the one-carbon metabolism and mitochondrial respiration pathways through crosstalk of H3K4me3 with H3pT11 during glucose depletion. This connection is important for protection of cells from oxidative damage. NUAK1 may play a crucial role in determining uptake of glucose or serine. Interestingly, the SESAME complex regulates transcription through phosphorylation of H3T11 using *de novo* serine under the growth media in yeast (7). Our data suggested that NUAK1 phosphorylates H3T11 by sensing availability of serine during glucose starvation.

## Experimental procedures

### Cell lines and tissue culture

HEK293 and HEK293T cells were purchased from the American Type Culture Collection and maintained in high-glucose DMEM (Gibco, 11995065) supplemented with 10% fetal bovine serum (Gibco A5670701) and 1% penicillin–streptomycin (Gibco, 15070063). Cells were cultured at 37 °C in a humidified incubator with 5% CO_2_. For glucose starvation experiments, cells were washed once withPBS and cultured in glucose-free DMEM (Gibco, 11966025) for the indicated times. Transfection of HEK293 was performed using a standard calcium phosphate precipitation method. Shortly, plasmids were added into 0.25 M CaCl2 and then mixed with same volume 2 X HBS buffer (1.5 mM Na2PO4, 280 mM NaCl, 10 mM KCl, 12 mM Glucose, and 50 mM HEPES, pH7). Then the plasmid-calcium phosphate mix was added into preseeded HEK293 cells.

To generate NUAK1 stably expressing cell lines, HEK293 cells were transfected with a 3 × Flag-tagged NUAK1 expression plasmid and FLAG-NUAK1 expressed cells were then selected with 500 μg/ml G418 for 14 to 21 days.

To generate stable NUAK1- and CXXC1-knockdown cell lines, HEK293 cells were transfected with a 3 × v single-guide RNA (sgRNA)/Cas9 all-in-one expression vector targeting NUAK1 (Genecopoeia, HCP223331-CG04-3), CXXC1 (Genecopoeia, HCP208799-CG12-3), or controls (Genecopoei, HCP220858-CG01-3) and were then selected with 500 μg/ml G418 for 14 to 21 days.

For SETD1A knockdown, lentivirus production was used. Briefly, sgRNA) sequences targeting SETD1A (GTCCTTGGGGCCAGAGATACAGG) were cloned into the lentiCRISPR v2 vector (Addgene, #52961). HEK293T cells were cotransfected with the sgRNA-containing lentiCRISPR v2-SETD1AsgRNA (for SETD1A-KD virus) or empty LntiCRISPR v2 vector (for control-KD virus shown in Fig. S2), psPAX2 (Addgene, #12260), and pMD2.G (Addgene, #12259) to obtain the lentiviral particles. HEK293 cells were then infected with the resulting lentiviruses. The cell culture medium was replaced with 10% fetal bovine serum containing DMEM 12 h after infection, and infected cells were selected with 100 μg/ml puromycin for 7 days.

### Cytosolic/nuclear/histone protein extraction

Cell pellets were suspended in five times of cell pellet volumes of ice-cold extraction buffer I (10 mM HEPES, pH 7.9, 1.5 mM MgCl_2_, 10 mM KCl, 1 mM DTT, 1 mM PMSF, and protease inhibitor cocktail (MilliporeSigma, 1836170001)) and were incubated on ice for 10 min. The cytoplasmic extracts were obtained from the supernatant after the centrifugation at 5000 rpm for 5 min at 4 °C. The remaining pellets were resuspended in three times of pellet volumes of extraction buffer II (20 mM HEPES, pH 7.9, 1.5 mM MgCl_2_, 420 mM NaCl, 0.1 mM EDTA, 25% (v/v) glycerol, 1 mM DTT, 1 mM PMSF, and protease inhibitor cocktail), followed by incubation at 4 °C for 1 h with gentle agitation and were then centrifuged at 14,000 rpm for 20 min at 4 °C. Nuclear extracts were obtained from the supernatants. The remaining pellets were further resuspended in three times of pellet volumes of 1 M HCl and incubated at 4 °C for 20 min to extract histones. Following centrifugation at 14,000 rpm for 20 min at 4 °C, the supernatants were transferred to fresh tubes, and histones were precipitated by adding three volumes of 100% TCA. Samples were incubated at 4 °C for 20 min and centrifuged at 14,000 rpm for 20 min at 4 °C, and histone pellets were washed with ice-cold acetone and air-dried. The cytoplasm and nuclear extracts were used for Western blot. Antibodies used for Western blots are described as follows: anti-H3pT11 (Abcam, ab5168), anti-NUAK1(Cell Signaling Technology, 4458S), anti-H3 (Abcam, ab1791), anti-β-actin (Cell Signaling Technology, 3700S), anti-FLAG (Sigma-Aldrich, F1804), anti-SETD1A (Bethyl Laboratories, A300–289A), anti-CXXC1 (Abcam, ab198977), anti-DPY30 (Bethyl Laboratories, A304-296A), anti-p53(Proteintech, 10442-1-AP; Santa Cruz, sc-126), anti-p-p53(S15) (Santa Cruz, sc-51690) and anti-HK2 (Proteintech, 66974-1-Ig). For Western blots and detection, NC (Nitrocellulose) membranes (Bio-rad, 1620112) and ECL substrate (Bio-rad, 1705061) were used.

### Flag purification

Cytoplasmic or nuclear extracts were adjusted to a 300 mM NaCl concentration by addition of 5 M NaCl or extraction buffer I, as appropriate and were subjected to ultracentrifugation at 45,000 rpm for 1.5 h at 4 °C to remove insoluble material. Preequilibrated anti-FLAG M2 agarose beads (MilliporeSigma, A2220) were incubated with the extracts for overnight at 4 °C. The beads were then washed with FLAG wash buffer (10 mM HEPES, pH 7.9, 1.5 mM MgCl_2_, 300 mM NaCl, 10 mM KCl, and 0.5% Triton X-100) three times. FLAG-tagged proteins were eluted using elution buffer (10 mM HEPES, pH 7.9, 1.5 mM MgCl_2_, 150 mM NaCl, 0.05% Triton X-100, 400 μg/ml 3 × FLAG peptides, protease inhibitor cocktail). Eluted proteins were analyzed by silver staining, Western blotting, and mass spectrometry.

### RNA extraction, complementary DNA (cDNA) synthesis, and qPCR

Total RNA was extracted from cells cultured in 6-well plates using TRIzol reagent (Thermo Fisher Scientific, 15596018) according to the manufacturer’s instructions. Briefly, cells were lysed in 1 ml TRIzol reagent per well, followed by addition of 200 μl chloroform. Samples were vortexed for 15 s and centrifuged at 4 °C for 15 min. The aqueous phase was transferred to a fresh tube, mixed with 500 μl isopropanol, vortexed for 15 s, and centrifuged at 4 °C for 10 min to precipitate RNA. The RNA pellet was washed once with 75% ethanol, centrifuged at 4 °C for 5 min, and resuspended in RNase-free water. Complementary DNA (cDNA) was synthesized using the Applied Biosystems High-Capacity RNA-to-cDNA Kit (Applied Biosystems,4387406) according to the manufacturer’s protocol. Reverse transcription reactions were incubated at 37 °C for 1 h and terminated by heating at 95 °C for 5 min. Quantitative PCR was performed using SYBR Green–based detection. Newly synthesized cDNA was diluted with nuclease-free water, and qPCR reactions were assembled by mixing 2.5 μl diluted cDNA, 2.5 μl gene-specific primers (5 μM, target HK2-1:forward: 5′-ctggctctggatcttggagg-3′, reverse: 5′-ctcagggatggcatagatctga-3′; HK2-2, forward: 5′- gtttgagaagatgatcagtggga-3′, reverse: 5′-tcaatgtctgagatgtctttggt-3′; PCK2-1, forward: 5′- atcacgaggtcaggagttgg-3′, reverse: 5′- cccgggttcaagcaattctc -3′; PCK2-2, forward: 5′-ccagaaaaggcgagtggatg-3′, reverse: 5′-ctcatctgcccacccaacta-3′), and 5 μl SYBR Green master mix (Quantabio, 95074-05K) in 96-well plates. Reactions were run on a CFX Real-Time PCR Detection System (Bio-Rad) using standard cycling conditions. Relative gene expression levels were calculated using the ΔΔCt method and normalized to internal control genes (*GAPDH*: forward: 5′-ggagcgagatccctccaaaat-3′, reverse: 5′-ggctgttgtcatacttctcatgg-3′).

### Mass spectrometry analysis

TCA-precipitated protein samples were analyzed independently by multidimensional protein identification technology (MudPIT). Briefly, protein pellets were resuspended in 100 mM Tris-HCl (pH 8.5) containing 8 M urea to denature proteins. Samples were reduced and alkylated prior to sequential digestion with recombinant LysC (Promega) followed by trypsin (Promega). Digestion reactions were quenched by addition of formic acid (FA) to a final concentration of 5%. Peptide samples were pressure-loaded onto 100 μm fused silica microcapillary columns packed sequentially with 9 cm of reverse-phase material (Aqua, Phenomenex), 3 cm of 5-μm strong cation exchange material (Luna, Phenomenex), and 1 cm of 5-μm C18 reverse-phase material. The loaded columns were placed in-line with an Agilent 1200 G1311 quaternary HPLC system. Eluting peptides were electrosprayed directly into an Orbitrap Elite mass spectrometer (Thermo Fisher Scientific) using a custom-built nano–LC electrospray ionization source by applying a distal voltage of 2.5 kV.

Full MS spectra were acquired over an m/z range of 400 to 1600, followed by data-dependent fragmentation in the ion trap (35% collision energy) of the first to fifth most intense ions from each full MS scan. Dynamic exclusion was enabled for 90 s. Mass spectrometer scan functions and HPLC solvent gradients were controlled using the Xcalibur data system (Thermo Fisher Scientific).

RAW data files were extracted into MS2 format using RawDistiller v1.0 (in-house developed software). RawDistiller settings were used to abstract MS1 scan profiles by Gaussian fitting and to implement dynamic offline lock mass correction using six background polydimethylcyclosiloxane ions as internal calibrants. MS/MS spectra were searched using ProLuCID with a mass tolerance of 10 ppm for both precursor and fragment ions. Trypsin specificity was required at both peptide termini during database searching against a combined protein database consisting of 81,592 human protein sequences (NCBI release 2020-11-23) and 426 common contaminant proteins, including keratins, immunoglobulins, and proteolytic enzymes.

To estimate false discovery rates, each protein sequence in the database was randomized while preserving amino acid composition and sequence length, and the resulting shuffled sequences were appended to the target database, yielding a total of 163,860 protein entries. Carbamidomethylation of cysteine residues (+57.0125 Da) was set as a static modification, and oxidation of methionine residues (+15.9949 Da) was included as a variable modification.

Peptide-spectrum matches were filtered and assembled using DTASelect v1.9 with the following criteria: a minimum DeltaCn value of 0.08; minimum XCorr values of 1.8, 2.1, and 2.5 for singly-, doubly-, and triply charged spectra, respectively; and a minimum peptide length of 7 amino acids. Results from individual runs were merged and compared using CONTRAST. Across combined replicate analyses, proteins were required to be identified by at least two unique peptides and/or two spectral counts. Proteins that were subsets of other identified proteins were removed using the parsimony option in DTASelect.

Proteins identified by identical peptide sets, including at least one peptide unique to distinguish protein isoforms, were grouped together, and a single representative accession number was assigned to each protein group. Normalized spectral abundance factor analysis was performed using normalized spectral abundance factor 7 to generate final reports of all detected peptides and nonredundant proteins across all runs.

### RNA sequencing

RNA sequencing libraries were prepared using the TruSeq Stranded mRNA Library Prep Kit (Illumina, 20020595) according to the manufacturer’s instructions. Libraries were sequenced on an Illumina HiSeq platform to generate paired end reads of 75 bp. All RNA-seq experiments were performed with biological triplicates.

### RNA-sequencing analysis

Raw reads were demultiplexed into Fastq format allowing up to one mismatch using Illumina bcl-convert 3.10.5. Reads were aligned to UCSC genome hg38 with STAR aligner (version 2.7.3a), using Ensembl 106 gene models. TPM values were generated using RSEM (version v1.3.0). false discovery rate cut-off of 0.05 was used to determine statistical significance. Differential gene expression analysis was performed by comparing expression levels between experimental conditions. GO and KEGG pathway enrichment analyses were conducted using the GO–KEGG analysis tools in Hiplot Pro, a web-based platform for biomedical data analysis and visualization.

### Kinase assay

*In vitro* kinase assays were performed by incubating 50 ng of recombinant GST–NUAK1 (Sigma-Aldrich, SRP5237) with 1 μg of recombinant histone octamer (EpiCypher, SKU: 16-0001) in NEB kinase buffer (New England Biolabs, 101640-774) in the presence or absence of 1 mM ATP (Roche, 09744363103) at 30 °C for 2 h and then terminated by adding SDS sample buffer, followed by boiling at 95 °C for 5 min prior to applying for SDS–PAGE and Western blots.

### Quantification and statistical analysis

Statistical analyses were performed using GraphPad Prism 10 software and Excel. The sample size was indicated in the figure legends. Data was presented as means ± SEM. Student’s *t* test with unpaired and two tail was applied to determine significant differences between the two samples. Differences were considered significant for *p*-values <0.05. For blotting bends, Fiji was used to calculate the band intensity and normalization.

## Data availability

Original RNA-sequencing data associated with this article are available from NCBI GEO with GSE313047. https://www.ncbi.nlm.nih.gov/geo/query/acc.cgi?acc=GSE313047. Original mass spectrometry proteomics data are available at the ProteomeXchange Consortium with the dataset identifier accession: PXD072152 *via* the MassIVE repository. All original data will be accessible from the Stowers Original Data Repository at. https://www.stowers.org/research/publications/LIBPB-2607.

## Supporting information

This article contains supporting information.

## Conflict of interest

The authors declare that they have no conflicts of interest with the contents of this article.

## References

[1] Andringa K.K., Coleman M.C., Aykin-Burns N., Hitchler M.J., Walsh S.A., Domann F.E. (2006). Cancer Res..

[2] Ren Y., Shen H.M. (2019). Critical role of AMPK in redox regulation under glucose starvation. Redox Biol..

[3] Bluemel G., Planque M., Madreiter-Sokolowski C.T., Haitzmann T., Hrzenjak A., Graier W.F. (2021). PCK2 opposes mitochondrial respiration and maintains the redox balance in starved lung cancer cells. Free Radic. Biol. Med..

[4] Suganuma T., Hassan H., Swanson S.K., Laxman S., Workman J.L. (2026). Altered transposon element-derived genes distort oxygen-free radical scavenger systems in FXD. NAR Mol. Med..

[5] Suganuma T., Workman J.L. (2018). Chromatin and metabolism. Annu. Rev. Biochem..

[6] Suganuma T., Workman J.L. (2023). Chromatin balances cell redox and energy homeostasis. Epigenetics Chromatin.

[7] Li S., Swanson S.K., Gogol M., Florens L., Washburn M.P., Workman J.L. (2015). Serine and SAM responsive complex SESAME regulates histone modification crosstalk by sensing cellular metabolism. Mol. Cell.

[8] Oh S., Lee J., Swanson S.K., Florens L., Washburn M.P., Workman J.L. (2020). Yeast Nuak1 phosphorylates histone H3 threonine 11 in low glucose stress by the cooperation of AMPK and CK2 signaling. Elife.

[9] Oh S., Suganuma T., Gogol M.M., Workman J.L. (2018). Histone H3 threonine 11 phosphorylation by Sch9 and CK2 regulates chronological lifespan by controlling the nutritional stress response. eLife.

[10] Escalona E., Munoz M., Pincheira R., Elorza A.A., Castro A.F. (2020). Cytosolic NUAK1 enhances ATP production by maintaining proper glycolysis and mitochondrial function in cancer cells. Front. Oncol..

[11] Palma M., Riffo E., Farias A., Coliboro-Dannich V., Espinoza-Francine L., Escalona E. (2023). NUAK1 coordinates growth factor-dependent activation of mTORC2 and Akt signaling. Cell Biosci..

[12] Suzuki A., Kusakai G., Kishimoto A., Lu J., Ogura T., Esumi H. (2003). ARK5 suppresses the cell death induced by nutrient starvation and death receptors via inhibition of caspase 8 activation, but not by chemotherapeutic agents or UV irradiation. Oncogene.

[13] Suzuki A., Kusakai G., Shimojo Y., Chen J., Ogura T., Kobayashi M. (2005). Involvement of transforming growth factor-beta 1 signaling in hypoxia-induced tolerance to glucose starvation. J. Biol. Chem..

[14] Wang H., Helin K. (2025). Roles of H3K4 methylation in biology and disease. Trends Cell Biol..

[15] Shilatifard A. (2012). The COMPASS family of histone H3K4 methylases: mechanisms of regulation in development and disease pathogenesis. Annu. Rev. Biochem..

[16] Kim J., Kim J.A., McGinty R.K., Nguyen U.T., Muir T.W., Allis C.D. (2013). The n-SET domain of Set1 regulates H2B ubiquitylation-dependent H3K4 methylation. Mol. Cell.

[17] Suzuki A., Kusakai G., Kishimoto A., Lu J., Ogura T., Lavin M.F. (2003). Identification of a novel protein kinase mediating Akt survival signaling to the ATM protein. J. Biol. Chem..

[18] Sonoda H., Aoki J., Hiramatsu T., Ishida M., Bandoh K., Nagai Y. (2002). A novel phosphatidic acid-selective phospholipase A1 that produces lysophosphatidic acid. J. Biol. Chem..

[19] Wu C., Dai C., Li X., Sun M., Chu H., Xuan Q. (2022). AKR1C3-dependent lipid droplet formation confers hepatocellular carcinoma cell adaptability to targeted therapy. Theranostics.

[20] Wilhelm M., Kukekov N.V., Schmit T.L., Biagas K.V., Sproul A.A., Gire S. (2012). Sh3rf2/POSHER protein promotes cell survival by ring-mediated proteasomal degradation of the c-Jun N-terminal kinase scaffold POSH (Plenty of SH3s) protein. J. Biol. Chem..

[21] Pranzini E., Pardella E., Muccillo L., Leo A., Nesi I., Santi A. (2022). SHMT2-mediated mitochondrial serine metabolism drives 5-FU resistance by fueling nucleotide biosynthesis. Cell Rep..

[22] Escos M., Latorre P., Hidalgo J., Hurtado-Guerrero R., Carrodeguas J.A., Lopez-Buesa P. (2016). Kinetic and functional properties of human mitochondrial phosphoenolpyruvate carboxykinase. Biochem. Biophys. Rep..

[23] Haidurov A., Zheltukhin A.O., Snezhkina A.V., Krasnov G.S., Kudryavtseva A.V., Budanov A.V. (2025). p53-regulated SESN1 and SESN2 regulate cell proliferation and cell death through control of STAT3. Cell Commun. Signal..

[24] Clark D.W., Mitra A., Fillmore R.A., Jiang W.G., Samant R.S., Fodstad O. (2008). NUPR1 interacts with p53, transcriptionally regulates p21 and rescues breast epithelial cells from doxorubicin-induced genotoxic stress. Curr. Cancer Drug Targets.

[25] Du C., Liu C., Yu K., Zhang S., Fu Z., Chen X. (2024). Mitochondrial serine catabolism safeguards maintenance of the hematopoietic stem cell pool in homeostasis and injury. Cell Stem Cell.

[26] Roberts D.J., Miyamoto S. (2015). Hexokinase II integrates energy metabolism and cellular protection: Akting on mitochondria and TORCing to autophagy. Cell Death Differ..

[27] Vincent E.E., Sergushichev A., Griss T., Gingras M.C., Samborska B., Ntimbane T. (2015). Mitochondrial phosphoenolpyruvate carboxykinase regulates metabolic adaptation and enables glucose-independent tumor growth. Mol. Cell.

[28] Bergstrand S., Bohm S., Malmgren H., Norberg A., Sundin M., Nordgren A. (2020). Biallelic mutations in WRAP53 result in dysfunctional telomeres, Cajal bodies and DNA repair, thereby causing Hoyeraal-Hreidarsson syndrome. Cell Death Dis..

[29] Mahmoudi S., Henriksson S., Corcoran M., Mendez-Vidal C., Wiman K.G., Farnebo M. (2016). Wrap53, a natural p53 antisense transcript required for p53 induction upon DNA damage. Mol. Cell.

[30] An S., Yao Y., Hu H., Wu J., Li J., Li L. (2023). PDHA1 hyperacetylation-mediated lactate overproduction promotes sepsis-induced acute kidney injury via Fis1 lactylation. Cell Death Dis..

[31] Wang H.P., Singh S., Wong L.C., Hsu C.J., Li S.C., Lee S.J. (2025). Lacosamide is a novel drug that improves AGTPBP1 knockout-mediated impairment of neuronal and dopaminergic function. Mol. Neurobiol..

[32] Lopez-Domenech G., Serrat R., Mirra S., D'Aniello S., Somorjai I., Abad A. (2012). The Eutherian Armcx genes regulate mitochondrial trafficking in neurons and interact with Miro and Trak2. Nat. Commun..

[33] Fenton A.R., Jongens T.A., Holzbaur E.L.F. (2021). Mitochondrial adaptor TRAK2 activates and functionally links opposing kinesin and dynein motors. Nat. Commun..

[34] DeWaal D., Nogueira V., Terry A.R., Patra K.C., Jeon S.M., Guzman G. (2018). Hexokinase-2 depletion inhibits glycolysis and induces oxidative phosphorylation in hepatocellular carcinoma and sensitizes to metformin. Nat. Commun..

