# Supplementary material for: Crosstalk between NUAK1 and the SETD1A complex regulates glycolysis and gluconeogenesis upon glucose deprivation[image]
Source: J Biol Chem. 2026 Sep 23;302(11):113598. doi: 10.1016/j.jbc.2026.113598 (PMC13696809; doi:10.1016/j.jbc.2026.113598)

**Crosstalk between NUAK1 and the SETD1A complex regulates glycolysis and gluconeogenesis upon glucose deprivation**

Zhen Wang[1], Yan Hao[1], Shiyuan Chen[1], Laurence Florens[1], Jerry L. Workman[1,2], and Tamaki Suganuma[1,3],

[1]Stowers Institute for Medical Research, Kansas City, MO 64110, USA

[2]Corresponding: jlw@stowers.org

[3]Corresponding and lead contact: tas@stowers.org

**Supporting Information**

Supporting Figures 1-4 (Fig. S1-S4)

Supporting Figure legends (for Fig. S1-S4)

Supporting Tables 1-3 (Table S1-S3, Excel Files)

Supporting Table legends (for Table S1-S3)

**Supporting Information**

**Supporting Figures and Table Legends**

**Supporting Figure S1. Glucose depletion changes expression levels of genes in HEK293 cells**

Volcano plot shows differentially expressed (DE) genes upon glucose depletion for 36 hours in control-KD HEK293 cells. DE genes were selected with p-value < 0.05 and fold change > 2 or < -2. Red dots indicate up-regulated genes (n=1455) and blue dots indicate down-regulated genes (n=2002). Representative genes were indicated: *LIPH*, *lipase H*; *AKR1C3*, *aldo-keto reductase family 1 member C3*; *SH3RF2*, *SH3 domain containing ring finger 2*; *SHMT2*, serine hydroxymethyltransferase 2, *MTHFD2*, *methylenetetrahydrofolate dehydrogenase (NADP+ dependent) 2, methenyltetrahydrofolate cyclohydrolase*; *PCK2*, *phosphoenolpyruvate carboxykinase 2, mitochondrial*; *SESN2*, *sestrin 2*; *NUPR1*, *nuclear protein 1, transcriptional regulator*.

**Supporting Figure S2. Loss of NUAK1 induces apoptosis**

**A and B.** ROS-ID-stained ROS-positive living cells (DAPI positive) cultured in the media with or without glucose and pyruvate were measured using a flow cytometer. Histograms display representative ROS-ID intensity and the cell numbers in each sample. Negative controls were prepared from control-KD cells cultured in the presence of glucose treated with 5 mM N-acetyl-L-cysteine (ROS inhibitor). Positive controls were prepared from control-KD cells cultured in the presence of glucose treated with 500 mM pyocyanin (ROS inducer). The median fluorescence intensity (MFI) in each control-KD and NUAK1-KD cells (each n = 4, p, p-value) was measured. Bar graphs display fold changes of the average MFI in each sample ± SD compared between presence or absence of glucose in each control-KD and NUAK1-KD cells (upper two panels), and between control-KD and NUAK1-KD cells in each presence or absence of glucose (lower two panels) (B). **C**. Quantification of apoptotic cells (stained with Annexin V) (n=3, p, p-value) of control-KD and NUAK1_KD cells cultured in the presence or absence of glucose for 36 hours.

**Supporting Figure S3. SETD1A expressions were examined by Western blot**

Nuclear extracts from SETD1A-knockdown and control-knockdown HEK293 cells cultured in the presence (+) or absence (-) of glucose for 36 hours were analyzed by Western blots. β-actin was used as loading control.

**Supporting Figure S4. NUAK1 is important for cell survival**

Graph displays populations (percentage) of the live Annexin V-negative/DAPI-negative cells compared to total live cells in each control-KD, NUAK1-KD, and CXXC1-KD cells cultured in the presence or absence of glucose. (n=3), p, p-value.

**Supporting Table S1. Genes with modified transcripts levels upon glucose depletion and NUAK1-KD.**

Differentially expressed genes (p-val < 0.05) found in RNA-sequencing by comparisons between NUAK1-KD and control-KD in each presence (+) or absence (-) of glucose culture condition are listed (Fig. 2B). Genes with changed expression levels upon glucose depletion and/or NUAK1-KD vs controls found in Go analysis clustered by KEGG pathways (Fig. 2C) or biological processes (BP) (Figs. 2D-2F) are analyzed by RNA-seq data are listed.

**Supporting Table S2. Genes with modified transcripts levels upon glucose depletion were compared between NUAK1-KD and SETD1A-KD**

Differentially expressed genes (p-val < 0.05) found in RNA-sequencing by comparisons between SETD1A-KD and control-KD in each presence (+) or absence (-) of glucose culture condition are listed (Fig. 4B). The genes encoding proteins regulating DNA damage response and signal transduction by p53 class mediator are highlighted in the gene list corresponding to cluster 6 in Fig. 4B. Differentially expressed genes (p-val < 0.05) by comparisons between SETD1A-KD, NUAK1-KD, and control-KD in the absence of glucose are listed (Fig. 4C). Genes with reduced expressions upon glucose depletion in NUAK1-KD (cluster 2 in Fig. 2B), SETD1A-KD (cluster 6 in Fig. 4B), and in both NUAK1-KD and SETD1A-KD (cluster 10 in Fig. 4C) were compared. The common genes (124 genes) in these comparisons shown in 3-venn diagram in Fig. 4D are listed. Genes with significantly changed transcripts levels (p-val < 0.05) upon glucose depletion

compared between NUAK1-KD, SETD1A-KD, and control-KD corresponding to Fig. 4D (124 genes) found in Go analysis clustered by biological processes (BP) are listed (Fig. 4E).

**Supporting Table S3. Genes were regulated by glucose starvation, NUAK1-KD and SETD1A-KD**

Genes with changed transcripts upon glucose depletion SETD1A-KD, and control-KD found in GO analysis clustered by biological processes (BP) are listed (Fig. 5D). Gene expression with FPKM in Cluster 6 and 7 were shown in table S3B.

## Supporting Experimental procedures

### ROS detection (ROS-ID)

Control-KD and NUAK1-KD cells were cultured in DMEM containing 10% FBS in the presence or absence of glucose and sodium pyruvate for 36 h. Cells were then harvested and stained with ROS-ID dye (ENZ-51011, Enzo Life Sciences) according to the manufacturer's instructions, followed by DAPI staining at 2 μg /mL to exclude dead cells. Control-KD cells were treated with N-acetyl-L-cysteine (5 mM) as negative controls or pyocyanin (500 μM) as positive controls before harvest. After exclusion of cell debris and DAPI-positive cells, ROS-ID fluorescence in live cells was measured using a ZE5 Cell Analyzer (Bio-Rad) with a 488-nm laser. ROS levels were quantified by median fluorescence intensity (MFI), and MFI values were normalized to control-KD cells cultured in the presence or absence of glucose.

### Annexin V/DAPI flow-cytometry analysis

Control-KD, NUAK1-KD, and CXXC1-KD cells were cultured in the presence or absence of glucose for 36 h. Cells were then harvested, washed with cold PBS, and resuspended in 100 μL Annexin V binding buffer. Cells were stained with Annexin V at a final concentration of 5 μg /mL and DAPI at a final concentration of 2 μg/mL for 15 min at room temperature in the dark. After incubation, 200 μL Annexin V binding buffer was added to each sample, and the stained cells were analyzed immediately by flow cytometry. Annexin V and DAPI fluorescence were detected using emission wavelengths of 530 nm and 465 nm, respectively. The percentage of viable cells was defined as the Annexin V-negative/DAPI-negative population.

Supplementary Figure S1

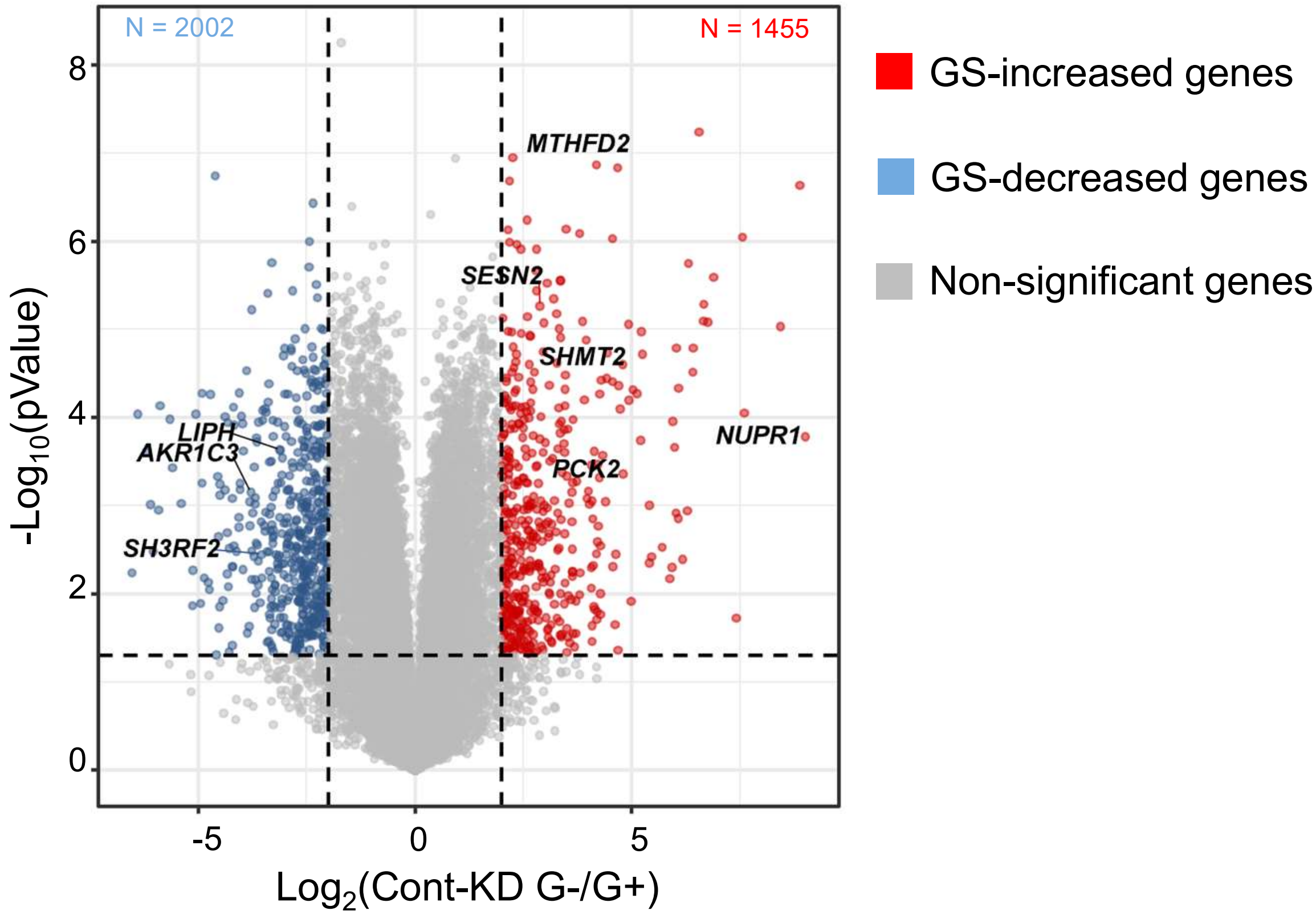

Supplementary Figure S2

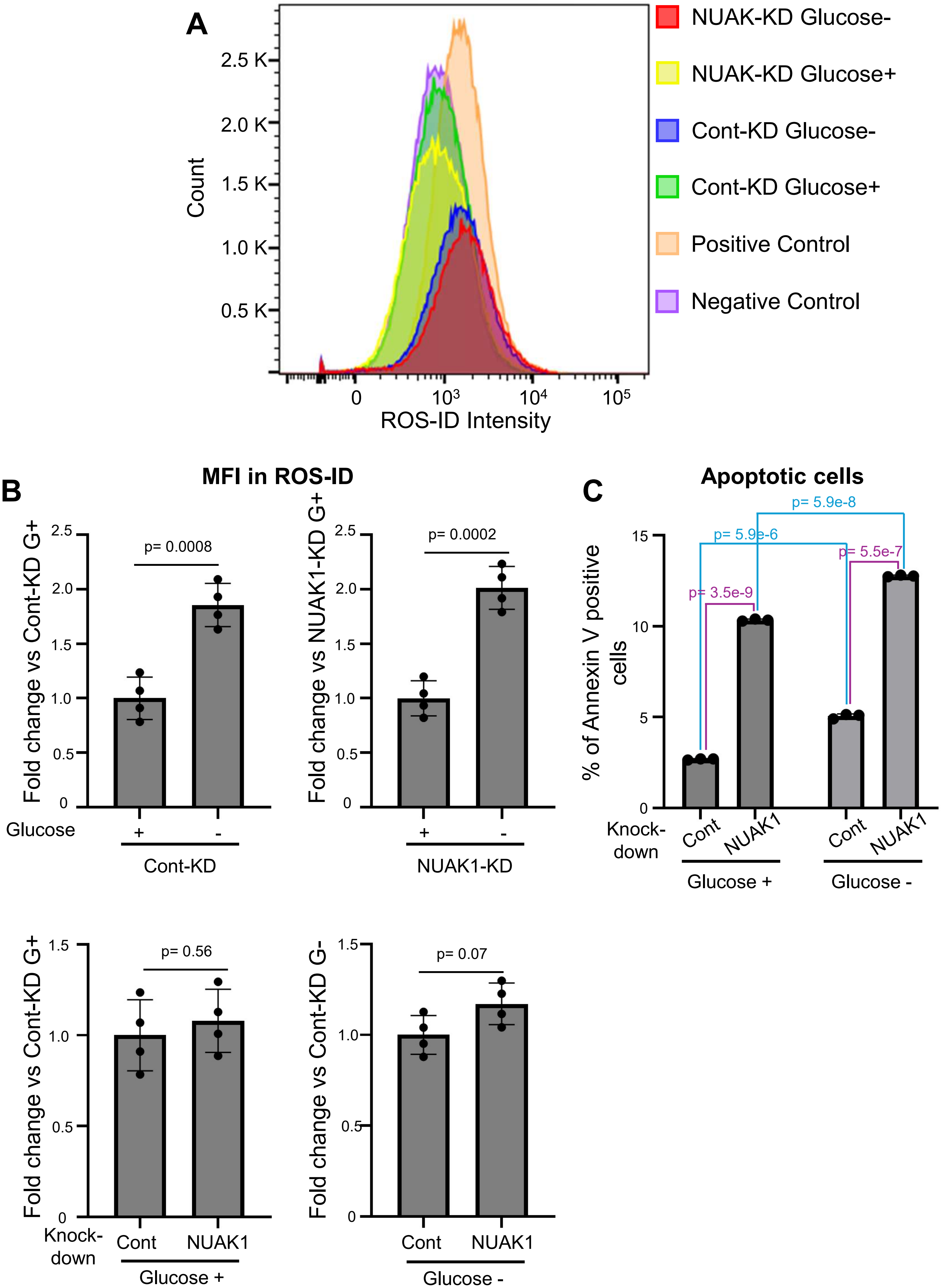

Supplementary Figure S3

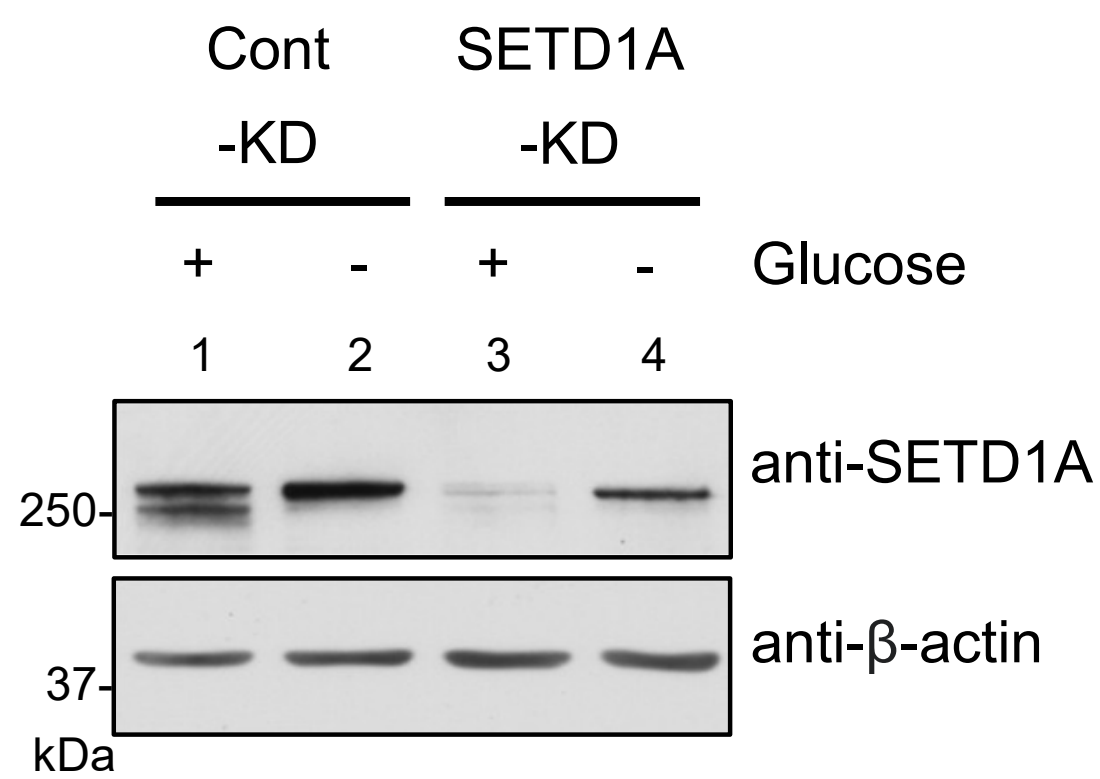

Supplementary Figure S4

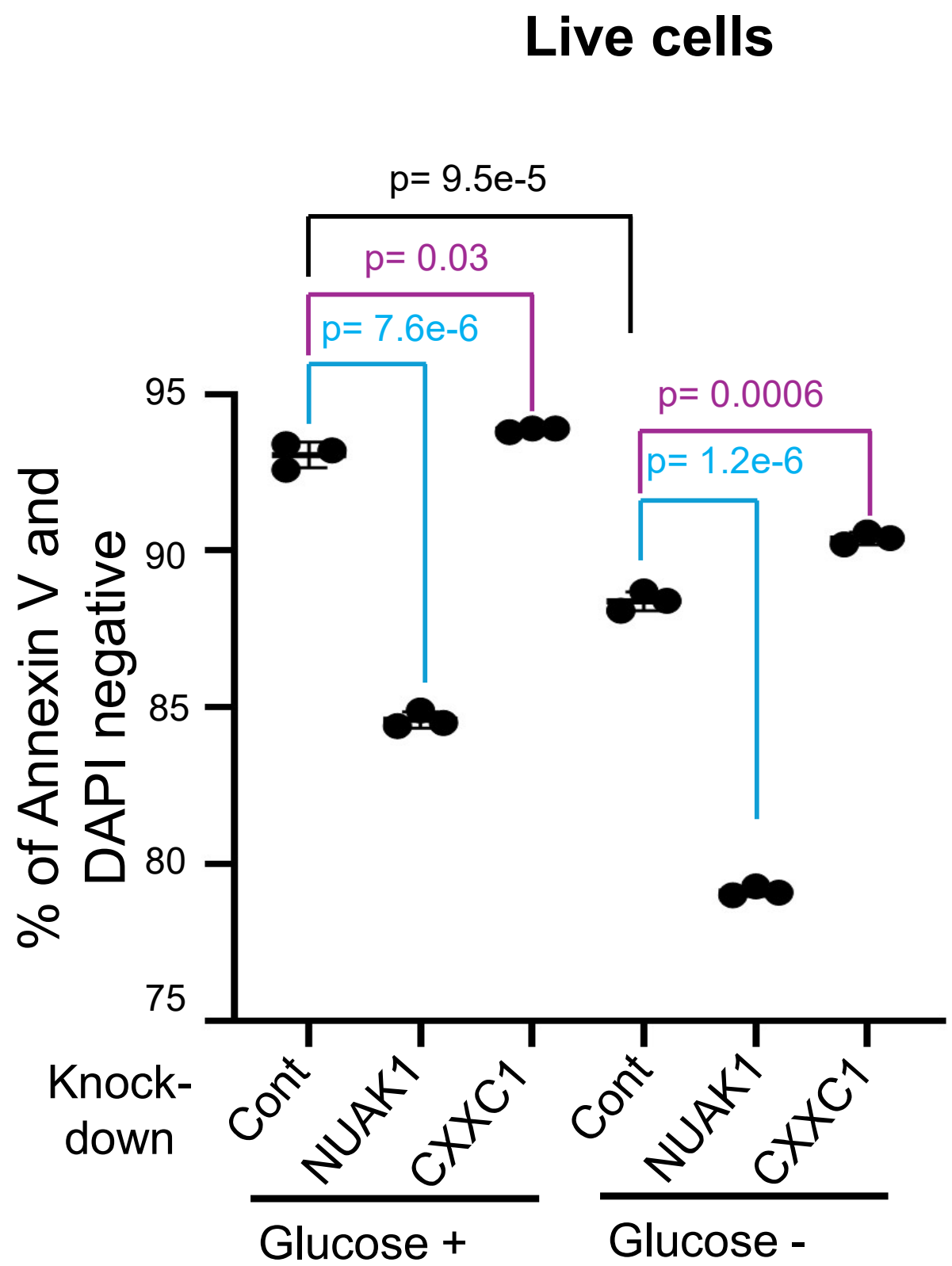

Supplement: Supplementary file 1 [file mmc4.pdf]
